# Mini-Review on Targeted Treatment of Desmoplastic Small Round Cell Tumor

**DOI:** 10.3389/fonc.2020.00518

**Published:** 2020-04-21

**Authors:** Tomas S. Bexelius, Ajla Wasti, Julia C. Chisholm

**Affiliations:** ^1^Children and Young People's Unit, Royal Marsden Hospital NHS Foundation Trust, Sutton, United Kingdom; ^2^Department of Women and Children Health at Karolinska Institutet, Stockholm, Sweden; ^3^Department of Pediatric Oncology, Seattle Children's Hospital, Seattle, WA, United States; ^4^Department of Pediatrics, University of Washington, Seattle, WA, United States; ^5^Division of Clinical Studies, The Institute of Cancer Research, London, United Kingdom

**Keywords:** anti-angiogenesis, pazopanib, multi-tyrosine kinase inhibitors, soft-tissue sarcoma, mTOR-inhibitors, MErT/EMT, targeted treatment

## Abstract

Desmoplastic small round cell tumor (DSRCT) is a devastating disease which most commonly affects adolescents, with a male predominance. Despite the best multimodality treatment efforts, most patients will ultimately not survive more than 3–5 years after diagnosis. Some research trials in soft-tissue sarcoma and Ewing sarcoma include DSRCT patients but few studies have been tailored to the specific clinical needs and underlying cytogenetic abnormalities characterizing this disease such as the typical EWSR1-WT1 gene fusion. Downstream activation of EWSR1-WT1 gene fusion includes signaling pathways of platelet-derived growth factor (PDGF), vascular endothelial growth factor (VEGF), and insulin growth factor (IGF)-1. Other biological pathways that are activated and expressed in DSRCT cells include endothelial growth factor receptor (EGFR), androgen receptor pathway, c-KIT, MET, and transforming growth factor (TGF) beta. Investigation of somatic mutations, copy number alterations (CNA), and chromosomes in DSRCT samples suggests that deregulation of mesenchymal-epithelial reverse transition (MErT)/epithelial-mesenchymal transition (EMT) and DNA damage repair (DDR) may be important in DSRCT. This mini review looks at known druggable targets in DSRCT and existing clinical evidence for targeted treatments, particularly multityrosine kinase inhibitors such as pazopanib, imatinib, and sorafenib alone or in combination with other agents such as mTOR (mammalian target of rapamycin) inhibitors. The aim is to increase shared knowledge about current available treatments and identify gaps in research to further efforts toward clinical development of targeted agents.

## Introduction

### Clinical Presentation and Current Treatment

DSRCT is a rare and aggressive soft-tissue sarcoma (1) that most commonly affects adolescent and young adult males (2, 3). DSRCT arises from serosal surfaces (4) and is molecularly characterized by translocation between Ewing sarcoma RNA binding protein 1 gene (EWSR1) and Wilms tumor suppressor gene (WT1). DSRCT often presents as advanced abdominal disease with multifocal deposits, peritoneal carcinomatosis and omental involvement with or without ascites (5). Patients may present with spread to the lymph nodes, liver and sometimes lungs (6).

Current treatment of DSRCT involves multimodal therapy, which may include intensive alkylator-based chemotherapy (7), cytoreductive surgery (8, 9) and involved field radiotherapy (10) with or without Hyperthermic Intraperitoneal Chemotherapy (HIPEC) at the time of cytoreductive surgery (11). In practice, not all patients are suitable for cytoreductive surgery and whole abdominopelvic radiotherapy may be highly morbid but with the use of intensity modulated radiation therapy (IMRT) toxicity, particularly hematological toxicity has reduced (12).

Other approaches such as immunotherapy (13) with B7-HR antibody clinical trial identifier (NCT02982941) or radio-immunotherapy targeting 131-8H9 clinical trials identifier NCT01099644) (13), but are outside of the scope of this review (**Table 2**).

Despite initial response to chemotherapy, DSRCT often progresses while patients are on treatment (6). The median time to progression on first-line treatment is short: in a UK study median time to progression was between 3.9 and 14.9 months depending on type of chemotherapy protocol used (2) and in a French study the progression-free survival was 11 months (14). The occasional long-term survivors are those with localized disease at presentation (15) and those who have complete surgical resection (14). The 5-year overall survival is dismal at 5–18% (14, 15) highlighting the urgent need for better systemic therapies for this highly aggressive tumor.

### Histopathology

DSRCT is thought to arise from a progenitor cell with multi-phenotypic potential (3, 5). Morphologically, DSRCT is a small blue round cell tumor with epithelioid and spindle cells surrounded by growth of connective/fibrous tissue, which is termed desmoplasia (5). By immunostaining DSRCT shows markers of neuronal, epithelial, and muscle differentiation (3).

## Molecular Characteristics OF DSRCT

DSRCT is characterized by the t(11;22)(p13;q12) chromosomal translocation (4) involving a fusion between the transcriptional activating domain of EWSR1 and the *WT1* gene, a tumor suppressor gene whose protein product is a transcriptional activator of genes involved in renal and gonadal differentiation and regulates the mesenchymal to epithelial transition seen in renal development (16). The EWSR1-WT1 gene fusion forms a chimeric protein acting as transcription factor with at least 35 known target genes, including PDGF (17), IGF-1 receptor, epidermal growth factor receptor (EGFR) and others such as c-MYC and fibroblast growth factor receptor (FGFR). This translocation and the resulting transcriptional changes are believed to be the major driver in DSRCT (3, 16).

There are limited data on other genetic aberrations in DSRCT although current national molecular profiling initiatives such as the planned NHS genomic medicine service for all newly diagnosed pediatric solid malignancies in children and young people and the Stratified Medicine Pediatrics (SM-Paeds, ISRCTN21731605) molecular profiling programme in relapsed solid tumors will in future provide further information (18). Among existing reports, one patient showed variants of unknown clinical significance in ARID1A and RUNX1 genes (19) Another study detected no mutations in a panel of 29 genes (including *MET, ALK* and *KIT*) evaluated in 24 cases of DSRCT (20) and in a large study of sarcomas analyzed by next generation or Sanger sequencing which included 9 DSRCT samples, few mutations were seen (21).

Jiang et al. (22) interrogated tumors from 10 DSRCT patients, who had received pre-treatment with chemotherapy, for somatic genetic mutations against a panel of genes known to be of importance in childhood cancer (*tumor suppressor and oncogenic drivers*) using a single-gene polymerase chain reaction (PCR) approach (22). Two of these 10 DSRCT cases had detectable somatic mutations. One case had a mutation in the *MET* gene coding for the c-Met tyrosine kinase, which has been classified as proto-oncogene acting on the hepatocyte growth factor/scatter factor (HGF/SF) (22). The second DSRCT case had a mutation in the gene for phosphatidylinositol-4,5-bisphosphate 3-kinase (PI3K) catalytic subunit alpha [PI3KCA] (22). PI3KCA acts on PI3K/AKT/mTOR pathway and is important for cell proliferation and tumor growth.

When whole-exome sequencing (WES) was used to interrogate DSRCT, 137 somatic mutations were found in 6 patients, but only two mutations were overlapping amongst cases (23). The authors subsequently classified the affected genes by biological function and more than a quarter of the mutated genes belonged to either DNA damage-response network (DDR) or genes that belong to mesenchymal-epithelial reverse transition (MErT), and EMT (epithelial-mesenchymal transition). Of interest, another WES study in DSRCT in one patient with DSRCT showed 12 somatic and 14 germline events in genes which were predominantly involved in mesenchymal differentiation (24) Poly(ADP-ribose) polymerase or PARP inhibitor has been suggested to be active in tumors with deficiency in DDR and in combination with DNA damaging agents (25). Currently there is a clinical trial underway for refractory pediatric solid tumors, which is investigating PARP inhibition using olaparib (26).

MErT/EMT is a common feature in malignant tumors and activation of these pathways is linked to increased invasiveness and the ability to metastasise, as has been described for sarcoma (27) There is no clinically available agent to address the MeRT/EMT switch in sarcoma. However, mesenchymal differentiation from tumor cells has been reported with use of trabectedin in Ewing sarcoma (28).

## Clinical Evidence for Targeted Agents in DSRCT

Published data and open clinical trials available in the clinical trial repositories investigating the effect of targeted treatment in DRSCT have been reviewed. Table 1 shows an overview of recently published reports including patients with DSRCT, and Table 2 summarizes clinical trials ongoing at the time of this submission. Currently targeted treatments are usually offered in instances where a patient with DSRCT has had disease progression despite first-line or second-line chemotherapy although better systemic therapies for front line treatment are urgently needed. A number of trials combine DSRCT with Ewing sarcoma and there is an absence of completed randomized studies in DSRCT owing to the rarity of the disease.

**Table 1:** Selected trials and case-reports including desmoplastic small round cell tumor.

**Phase of trial**	**Agent**	**N, total; (n), DSRCT patients**	**Response rate in DSRCT**	**Target and mechanism**	**Studies**
Phase I/II, case-report	Pazopanib	9 (9)	2 PR, 7 SD; >12 weeks	VEGFR, 1-3, c-KIT, PDGF-α	(29)
		1 (1)	1 SD for 6 months		(30)
		51 (2)	1 PR for 24 months		(31)
		29 (29)	16 SD, 1 PR, 1 CR; at 12 weeks		(32)
Case-report	Apatinib	1 (1)	1 PR for 4 months	Multi-receptor TKI inhibiting VEGFR-2	(33)
Case-series	Pazopanib and sirolimus	8 (1)	1 SD for 11 months	See above and mTOR inhibition affecting PI3K/AKT-RAS/RAF pathway	(34)
Case-report	Ridafirolimus	1 (1)	1 SD for 4 months	mTOR inhibition	(35)
Phase I	SU101	27 (2)	1 SD for 12 months	PDGF receptor inhibitor, downstream inhibition of EWRS1-WT1	(36)
Phase I/II	Imatinib mesylate	8 (1)	1 SD for 3 months	PDGF receptor inhibitor, c-KIT, VEGFR	(37)
		70 (10)	0 SD for 2 months		(38)
		7 (2)	1 SD for 11 months		(39)
Case-series	Sorafenib	9 (2 on sorafenib)	2 SD for 3–4 months	MAPK, VEGFR, PDGF, c-KIT	(35)
Case-series	Sunitinib	9 (2 on sunitib)	2 SD for 2–5 months	VEGFR	(40)
Case-report	Apatinib	1 (1)	1 PR for 4 months	Multi-receptor TKI inhibiting VEGFR-2	(33)
Phase I/II	Ganitumab	38 (16)	1 PR and 3 SD for >24 weeks	IGF-1R inhibitor blocking IGF-1 and IGF-2	(41)
Phase II	Cixutumumab and temsirolimus	20 (3)	2 SD for 5-6 months	IGF-1R inhibitor and mTOR inhibition	(42)
Case-report	Anlotinib	48 (1)	1 SD for 4 months	VEGFR, EGFR, PDGFR, and MET inhibitor	(43)
Case-series	Combined androgen blockade	6 (6)	1 PR, 2 SD for 3-4 months	Androgen receptor inhibition	(44)

*DSRCT, desmoplastic small round cell tumor; CR, complete response; PR, partial response; SD, stable disease; EGFR, endothelial growth factor receptor; VEGFR, vascular endothelial growth factor receptor; PDGFR, platelet-derived growth factor receptor; PI3K, hosphatidylinositol-4,5-bisphosphate 3-kinase, MAPK, mitogen-activated protein kinases*.

**Table 2:** Trials including patients with desmoplastic small round cell tumor.

**ClinicalTrials.gov Identifier**	**Status**	**Indication**	**Treatment**	**Comparison**
NCT01946529	Phase II	Ewing sarcoma and DSRCT	Temsirolimus, temozolomide and irinotecan	Vincristine, doxorubicin, cyclophosophamide (VDC) ifosfamide etoposide (IE)
NCT00055952	Phase II	Ewing's sarcoma, primitive neuroectodermal tumor, or DSRCT	Exatecan mesylate	N/A
NCT01532687	Phase II	Refractory soft tissue sarcoma	Gemcitabine hydrochloride with pazopanib	Gemcitabine hydrochloride with placebo
NCT03275818	Phase II	Desomid; DSRCT, ewing sarcoma age 18–80 years	Nab-paclitaxel	Single-arm
NCT03139331	Phase I	Relapsed or refractory sarcoma	Pazopanib, irinotecan, temozolomide	Single-arm
NCT02982486	Phase II	Non-resectable/metastatic sarcoma and endometrial carcinoma patients with somatic deficient mmr (mismatch repair)	Nivolumab and ipilimumab	Single-arm
NCT01956669	Phase II	Refractory solid tumors	Pazopanib	Single-arm
NCT03628131	Phase I/II	Combination chemotherapy with pazopanib in children and adolescents with relapsed/refractory solid tumors	Pazopanib + Ifosfamide, carboplatin, etoposid	Single-arm
NCT02048371	Phase II	SARC024: A blanket protocol to study oral regorafenib in patients with selected sarcoma subtypes	Regorafenib	Placebo
NCT04145349	Phase I/II	A randomized, open-label phase 1/2 study evaluating ramucirumab in pediatric patients and young adults with relapsed, recurrent, or refractory desmoplastic small round cell tumor	Ramucirumab, cyclophosphamide and vinorelbine	Cyclophosphamide, vinorelbine
NCT02982941	Phase I	Enoblituzumab (MGA271) in children with B7-H3-expressing Solid Tumors	Enoblituzumab	Single-arm
NCT01099644	Phase I	Intraperitoneal radioimmunotherapy With 131I-8H9 for patients With desmoplastic small round cell tumors and other solid tumors involving the peritoneum	131I-8H9	Single-arm
NCT02808650	Phase I	Prexasertib in treating pediatric patients with recurrent or refractory solid tumors	Prexasertib (LY2606368)	Single-arm
UMIN000025521^*^	Phase I	Phase I clinical study of oral olaparib in pediatric patients with refractory solid tumors	Olaparib	Single-arm

* *Clinical trials identifier from Japanese registry, protocol published Pubmed id 30684955*.

### Pazopanib

Pazopanib, an oral, second generation, multi tyrosine kinase inhibitor, has more clinical experience than most targeted agents in DSRCT. It acts mainly on c-KIT, VEGF 1,2 and 3, and PDGF receptor alpha and beta; and to a lesser extent FGFR 1 and 3 (45). *In vitro* studies showed highest affinity for the VEGF-1 of the VEGF receptors with inhibitory concentration (IC)50-values at nanomolar concentration (46). There is evidence of over-expression of VEGF in adult soft-tissue sarcoma (47).

In two small case series a late partial response was seen in one of two patients with DSRCT after 14 cycles of treatment (31) and in a second study by Frezza et al. partial response was seen in 2/9 patients after 12 weeks of follow up (29). In the largest study with DSRCT patients (*n* = 22) 16 patients had stable disease at 12 weeks of follow up with a median survival of 15.7 months, after having been on at least three different lines of chemotherapy (32).

These results suggest clinical activity of pazopanib in DSRCT. Interestingly, apatinib, another VEGFR-2 inhibitor had clinical activity (partial response) in a case of DSRCT treated with prior surgery and non-conventional treatment with a follow up time of 4 months (33). The anti-VEGF 2 monoclonal antibody ramicirumab is currently being evaluated in combination with backbone chemotherapy (cyclophosphamide and vinorelbine) in a randomized phase II study in DSRCT (NCT04145349; Table 2).

### Targeting PI3K/AKT/MTOR Pathway

The PI3K/AKT/mTOR pathway is involved in tumor development in pediatric sarcoma (48) and it appears to be constitutively activated in DSRCT, predominantly through TORC2 (49). The mTOR-inhibitor ridafirolimus achieved a 4-month stable response in a patient with DSRCT previously treated with chemotherapy and sunitinb with an overall survival of 38 months (35) and another heavily pretreated patient with DSRCT who was given temsirolimus achieved prolonged stabilization before progression (50).

As *PTEN* status is important for the outcome of mTOR inhibition (51), it is noteworthy that increased protein expression of *PTEN* was seen in 62% of 35 cases of DSRCT, although there was no significant difference in comparison with Ewing sarcoma (52).

### Combination of Pazopanib With MTOR-Inhibitor

Tumors treated with single targeted agents generally develop resistance. Eight patients with metastatic soft-tissue sarcoma were treated with sirolimus in combination with pazopanib (34). Following progression on single agent pazopanib. One case of DSRCT was included in this cohort; the patient received 12 months of single-agent pazopanib and subsequently 11 months of pazopanib-sirolimus combination showing stable disease on the combination for 11 months (34).

### Platelet-Derived Growth Factor Receptor Pathway in DSRSCT

PDGF is important for cell growth, proliferation and formation of blood vessels, all of which are pertinent for tumor growth. The five different isoforms of PDGF act on two different receptors; PDGF-R alpha and beta, which are tyrosine kinases that become auto-phosphorylated upon activation. The expression of PDGF-R alpha (39) and beta (53) been shown in DSRCT samples. PDGF can stimulate a quiescent fibroblast or smooth muscle to progress in the cell cycle leading to DNA replication and cell division (54).

The EWSR1-WT1 translocation gene product induces expression of endogenous PDGF-A in tumor cells from DSRCT but not from Ewing's sarcoma (17). The expression of PDGF-A may have a role in desmoplasia seen in DSRCT but a study done by Zhang et al. found an inverse association between PDGF-A expression and desmoplasia when analyzing patient DSRCT samples (53).

The first clinical trial targeting PDGFR in DSRCT was using a small molecule inhibitor called SU101 or leflunomide and resulted in one patient out of 2 with DSRCT experiencing stable disease for more than 12 months before disease progression (36) Lenflunomide is not being developed further as an anti-sarcoma agent, but has been used as an immunosuppressant for treatment of rheumatoid arthritis (55).

Patients with metastatic soft-tissue sarcoma with biomarker expression of PDGFR alpha were treated with the monoclonal antibody olarutumab. Prolonged event free survival with olarutamub in combination with doxorubin was demonstrated in a phase Ib/II study (56) but this result was not confirmed in the recent randomized phase III trial of doxorubicin +/– olarapumab (57)The clinical evidence in DSRCT is scarce and in the study by Bulbul et al. (52), PDGFR expression was not found in the 8 patients analyzed (52).

### Targeting PDGF Receptors With Imatinib Mesylate

Imatinib mesylate is a tyrosine kinase inhibitor developed for treatment of chronic myeloid leukemia, which targets both PDGFR alpha and beta, and KIT-kinase. Imatinib has been tested in 3 studies with 13 DSRCT patients (37–39) The clinical benefit was limited to 2 patients who achieved stable disease (1 each in study by Bonde et al. and Chao et al.). Chao et al. reported one patient whose tumor had both KIT and PDGFR alpha expression had stable disease for 10 months, whereas a second patient whose tumor lacked PDGFR alpha expression progressed within a month of starting treatment (39).

### Targeting VEGF-Receptors With Sorafenib

As a multi-receptor tyrosine kinase inhibitor acting on mitogen-activated protein kinases (MAPK), VEGFR-2, VEGFR-3, Flt-3, PDGFR beta and c-KIT, sorafenib may also be a potential treatment for DSRCT (58). A French registry study dedicated to off-label use in sarcoma reviewed all patients on targeted treatments including sorafenib (35) Two patients with DSRCT who were commenced on sorafenib showed progression free survival of 3–4 months.

A similar multi-TKI, regorafenib, acting primarily on VEGFRs and PDGFRs is currently in testing for desmoplastic small round cell tumor in a placebo-controlled trial (Table 2; NCT02048371).

### Targeting IGF-1 With Monoclonal Antibody

The rationale for treating with IGF-1 receptor antibody is 2-fold. First, it has shown to have an anti-angiogenic effect by suppressing VEGF in sarcoma models (59). Second, it has been shown to have the additive effect of counteracting excessive AKT phosphorylation caused by mTOR-inhibitors in experimental models of Ewing sarcoma and rhabdomyosarcoma (59) Hence, there is a rationale for treatment with IGF-1 receptor inhibitors both as standalone treatment and in combination with mTOR inhibitors (60).

An open-label phase II study treated patients with Ewing's family tumor and DSRCT with ganitumab, an anti-IGF-1 receptor antibody. Four of 16 DSRCT patients showed clinical benefit (either stable disease or partial response ≥ 24 weeks). There was a higher clinical benefit seen in DSRCT with a mean progression free survival of 19 months vs. 7.9 for Ewing's sarcoma (41).

Another approach has been to combine the anti-IGF-1 receptor antibody cixutumumab with mTOR inhibitor temsirolimus to overcome resistance that has been seen in single-agent treatment. This approach resulted in stable disease for two of three DSRCT patients for at least 5 months i (42).

### Androgen Receptors as Potential Targets in DSRCT

Gene expression data showed enrichment in androgen receptor pathway compared to other sarcomas, such as Ewing sarcoma and alveolar rhabdomyosarcoma (52) This gene expression was confirmed by protein expression. Confirming this observation, Fine et al. found androgen receptors in 10 out of 27 DSRCT patients and *in vitro* assay showed growth of tumor cells when stimulated with diihydrotestosterone, indicating that they are functional. Three out of 6 patients (with confirmed androgen receptor expression) were treated with androgen blockade and sustained stable disease from 3–6 month after progressing on conventional treatment (44).

### Targeting c-MET Kinase With Anlotinib

There is limited published evidence of using MET-inhibitors for DSRCT. However, the multi-TKI anoltinib that has activity against VEGFR, EGFR, and PDGFR, also acts as a MET inhibitor. There is a single case-report of a patient with intra-abdominal DSRCT treated with anlotinib who had stable disease for 4 months of treatment post chemotherapy and surgery (43).

## Summary

The real break-through for systemic treatment of DSRCT has yet to come, partly because there is a need to improve understanding the biological processes underpinning the pathophysiology of the disease. Better understanding of the downstream effects of the characteristic EWSR-1/WT1 chromosomal translocation, and accumulating information on the frequency of druggable somatic mutations in DSCRT will provide avenues for future exploration. At present there are no agents that target the product of the characteristic gene fusion itself.

The existing clinical data using multi TKIs suggests some role for agents such as pazopanib that target VEGRs although to date these agents have been used principally in patients with relapsed or advance disease and the observed effects have been predominantly disease stabilization rather than disease regression.

Combination treatment with a second small molecule inhibitor such as mTOR inhibition could be a way of overcoming inter- and intra tumor heterogeneity but as yet the role of TKIs in first line treatment has not been evaluated.

Obstacles that have been highlighted in this mini-review include lack of prospective trials with significant numbers of DSRCT patients and, limited access to molecular profiling at diagnosis and disease progression to guide the use of suitable targeted agents. Even when potential targetable genetic alterations are known, availability of and access to relevant clinical trials in young patients can be a significant limitation (61).

Some potential targets such as the MeRT/EMT switch and DNA repair are worthy of further exploration.

## Author Contributions

TB drafted the article, together with JC. TB and JC devised the concept for the mini-review along with AW. All authors revised and contributed to the final version of the manuscript. AW and JC contributed with critical revision of the manuscript throughout the process.

## Conflict of Interest

The authors declare that the research was conducted in the absence of any commercial or financial relationships that could be construed as a potential conflict of interest.
